# RECQL5 at the **I**ntersection of **R**eplication and **T**ranscription

**DOI:** 10.3389/fcell.2020.00324

**Published:** 2020-05-25

**Authors:** Zeid Hamadeh, Peter Lansdorp

**Affiliations:** ^1^Terry Fox Laboratory, British Columbia Cancer Research Centre, Vancouver, BC, Canada; ^2^Department of Genome Science and Technology, University of British Columbia, Vancouver, BC, Canada; ^3^Department of Medical Genetics, University of British Columbia, Vancouver, BC, Canada; ^4^European Research Institute for the Biology of Ageing, University of Groningen, Groningen, Netherlands

**Keywords:** RECQL5, RECQ5, genome stability, cancer, DNA replication stress, transcription, replication conflict, DNA damage repair

## Abstract

Maintenance of genome stability is essential to prevent the accumulation of DNA mutations that can initiate oncogenesis and facilitate tumor progression. Studies of DNA repair genes have revealed a highly dynamic and redundant network of genes and proteins responsible for maintaining genome stability. Cancer cells are often deficient in DNA repair, and the resulting genome instability decreases their fitness but also allows for more rapid evolution under selective pressure. Of particular interest for genome stability are the RecQ class of helicases. Five genes in this class, *RECQL1, BLM, WRN, RECQL4*, and *RECQL5*, are unique to mammals, as simpler eukaryotes and bacteria appear to have only one homolog, RecQ. The precise role of each of the five mammalian RecQ helicases remains to be determined. Whereas loss of function mutations of *BLM, WRN*, and *RECQL4* in humans are associated with specific diseases, *RECQL1* and *RECQL5* have not yet been associated with specific disorders. Mice deficient in Recql5 are more likely to develop cancer, and human cells deficient in RECQL5 display chromosomal instability and elevated sister chromatid exchange events, similar to cells deficient in any of the other RecQ helicases. Recent studies support the hypothesis that RECQL5 can resolve intermediate DNA repair structures resulting from the collision of DNA transcription and replication machinery. In this review, we aim to summarize current knowledge regarding RECQL5 in the context of DNA repair, replication, and transcription to help uncover the role of RECQL5 in the maintenance of genome stability.

## Introduction

**FIGURE 1:**
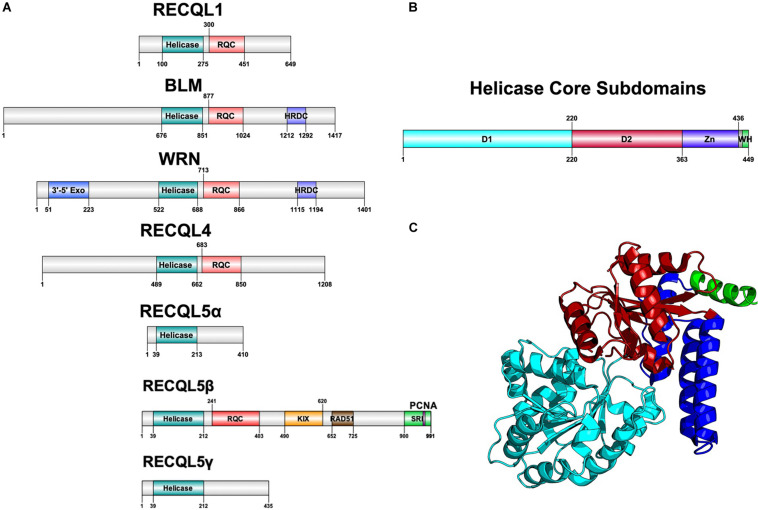
Structure of RecQ helicases. **(A)** Domain architecture of all five RecQ helicases, aligned by core helicase and RQC domains. **(B)** Subdomains of the core helicase domain of RECQL5. Zn refers to the Zn-binding domain and WH refers to the winged helix-like structure of RECQL5. **(C)** Cartoon structure diagram of the core helicase domain, colored by subdomain. Gene structure diagrams were designed using Domain Graph (DOG), and the protein structure was designed using PyMol with the crystal structure used in Newman et al. (2017). Data on gene structure was also retrieved from Croteau et al. (2014).

Helicases are a highly diverse class of motor proteins that use ATP to unwind or translocate strands of nucleic acids (Bernstein et al., 2010; Croteau et al., 2014). The RecQ helicases are one highly conserved class of DNA helicases from bacteria to complex eukaryotes and are known best for preventing inappropriate recombination (Bernstein et al., 2010). Bacteria and lower eukaryotes have only one RecQ ortholog, *RecQ*, whereas humans have five RecQ genes with a unique gene structure each, suggestive of functional divergence (Figure 1A).

All RecQ helicases share two common domains: the core helicase domain and the RecQ C-terminal (RQC) domain, which together make up the catalytic core of the enzyme (Figures 1B,C). Some members additionally contain a helicase and RNaseD C-terminal (HRDC) domain with a function that remains unclear but appears not to be essential for helicase activity (Newman et al., 2017). Within the core helicase domain, there are three subdomains, N and C terminal RecA-like core domains (D1 and D2) and a Zn^2+^-binding domain, followed by a winged helix (WH) responsible for interacting with DNA (Figures 1B,C). It is the catalytic core helicase domain that is responsible for unwinding dsDNA, translocating ssDNA, and, in some cases, remodeling of non B-DNA structures that may arise during transcription, repair, and replication (Wu, 2012).

Of the five RecQ helicases, *BLM, WRN*, and *RECQL4* are associated with specific diseases of marked premature aging and cancer predisposition such as Bloom Syndrome, Werner Syndrome, and Rothmund-Thompson Syndrome, respectively, whereas *RECQL1* and *RECQL5* remain to be associated with specific disorders (Bernstein et al., 2010). In a group of 50 mice deficient in the murine homolog of RECQL5, Recql5, nearly 50% developed cancer within 22 months compared to 6% in wildtype mice (Hu et al., 2007). Additionally, cells deficient in RECQL5 display a phenotype of chromosomal instability resulting in elevated sister chromatid exchange events (SCEs) and double-strand breaks (DSBs) similar to cells deficient in most of the other RecQ helicases (Hu et al., 2007). Unique to RECQL5 is a C-terminal domain consisting of multiple protein–protein interaction motifs that are believed to help RECQL5 regulate DNA repair intermediate structures resulting from the collision of DNA transcription and replication machinery (Khadka et al., 2016).

### Biochemical Characterization of RECQL5 Helicase

*RECQL5* was first cloned by Kitao et al. (1998) and was identified as a RecQ helicase based on homology with other characterized RecQ helicases. In humans, the gene is ubiquitously expressed in all tissues tested, with notably strong expression in the testis and pancreas (Kitao et al., 1998). *RECQL5* was mapped to chromosome 17q25 and found to be alternatively spliced in 19 variant forms, with three variant forms (α, β, and γ) being the most predominant (Kitao et al., 1998; Hu et al., 2007). The α and γ forms are less common variants that are truncated at the C-terminal and have only D1 and D2 helicase subdomains without the Zn^2+^-binding domain that is essential for helicase activity (Hu et al., 2007). Therefore, these truncated forms are deficient in helicase activity and only have a strand annealing function. The more common variant, RECQL5β (referred to hereinafter as RECQL5), is a 120 kDa protein with 991 amino acids containing all three core helicase subdomains and an extended C-terminal that is different from other RecQ helicases and contains several regions essential for specific protein–protein interactions (Figure 1A). It remains unclear to what degree different isoforms of RECQL5 play a role in different cell types.

Crystal structures of RECQL5 have revealed D1 and D2 helicase subdomains that are highly similar to other RecQ helicases, whereas a helical hairpin motif in the Zn^2+^-binding domain is significantly longer than that of any other RecQ helicase (Newman et al., 2017). Additionally, the C-terminal of RECQL5 lacks a winged helix immediately following the Zn^2+^ binding domain and instead has a positively charged alpha helix (Newman et al., 2017). Both of these unique structures in the core catalytic unit are believed to confer selectivity in the DNA-binding capacity of RECQL5 compared to other RecQ helicases. Newman et al. (2017) showed that this region contributes to a higher specificity in RECQL5 for non-duplex DNA such as ssDNA, hairpin loops in dsDNA, and forked DNA structures, all of which could occur as transcription intermediates.

Within the C-terminal of RECQL5 are two domains responsible for protein interactions (Newman et al., 2017). The kinase-inducible domain interacting (KIX) domain and Set2-Rpb1 interacting (SRI) domain were isolated from full-length RECQL5 constructs and were shown to be required for the interaction between RECQL5 and RNA polymerase II (RNAPII) (Table 1). Using purified proteins, Hu et al. (2007) demonstrated that RECQL5 is capable of binding and inhibiting RAD51-mediated D-loop formation, an interaction discovered to require a motif between residues 652 and 725. Electron microscopy revealed that RECQL5 can remove RAD51 from ssDNA in a reaction dependent on ATP hydrolysis and the ssDNA-binding protein, RPA. Several other stimulatory interactions are summarized in Table 1 and are discussed in further detail below.

**TABLE 1:** Protein–protein interactions reported for RECQL5.

**Protein**	**Region**	**Function**	**Reference**
FEN1	ND	Stimulates FEN1 endonuclease activity	Speina et al., 2010
Mre11	ND	Inhibits Mre11 activity	Zheng et al., 2009
NBS1	ND	ND	Zheng et al., 2009
PCNA	541-991	Promotes conjugation of PCNA with SUMO2	Kanagaraj et al., 2006
TOPO IIa	ND	Stimulates TOPOIIa decatenation activity	Ramamoorthy et al., 2012
TOPO IIIa	ND	ND	Shimamoto, 2000
RAD50	ND	ND	Zheng et al., 2009
RAD51	652-725	Disrupts RAD51 nucleofilaments	Hu et al., 2007
RNAPI	ND	ND	Urban et al., 2016
RNAP II	KIX, SRI	Inhibits the rate of RNAPII transcript elongation	Aygün et al., 2008; Kanagaraj et al., 2010
SWI/SNF complex	ND	ND	Zhou et al., 2010
WRN	ND	Stimulates helicase activity of WRN	Popuri et al., 2013

## Recql5 Gene Function

### Role of RECQL5 in Double-Stranded DNA Break Repair

**FIGURE 2:**
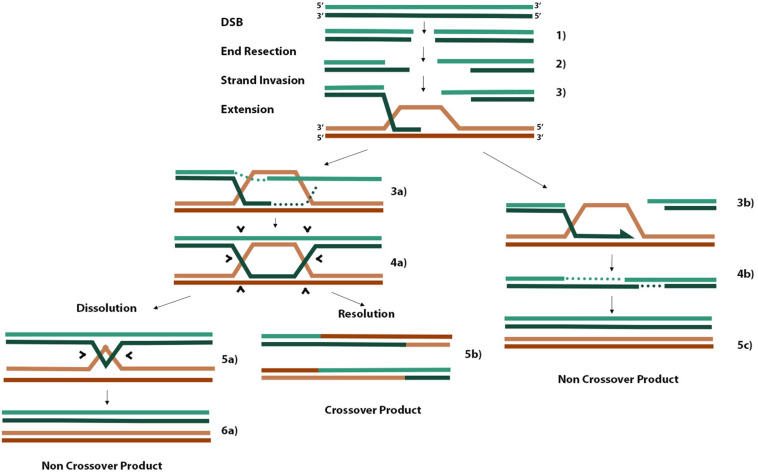
Homologous recombination schematic of different repair pathways. Three initial steps that are common to all pathways include end resection of 3′ overhangs, strand invasion of one or both overhangs with homologous donor DNA, and extension of annealed overhang **(steps 1–3)**. When only one resected end of the DSB performs invasion, a D-loop is formed, and extension proceeds by synthesis-dependent strand annealing where one overhang is extended until there is sufficient homology to hybridize with the other resected end, gaps are filled in, and nCOs are produced **(steps 3b, 4b, 5c)**. When the second resected end also hybridizes to the available strand in a D-loop, a dHJ is formed **(steps 3a, 4a)**. Processing of dHJs can proceed by promoting convergent migration of the structure until a small hemicatenane structure is formed **(step 5a)**, which can be cleaved by topoisomerases into an nCO product **(step 6a)**. Alternatively, asymmetric cleavage of the dHJ by non-specific resolvases can result in a CO product **(step 5b)**. The information from this figure was extracted from the works of Smith et al. (2007); West et al. (2016), and Rickman and Smogorzewska (2019).

When cells encounter DNA damage or replication stress that leads to a DSB, two main pathways are essential for faithful DNA repair. Non-homologous end joining (NHEJ) predominates during G1 because cells have yet to replicate their DNA and cannot access the redundancy of genetic material required as a template for faithful DNA repair by homologous recombination (HR) (Wright et al., 2018). There are three main steps in HR (Figure 2). Firstly, 3′ ssDNA overhangs are formed through end resection coordinated by the MRE11-RAD50-NBS1 complex at the DSB (Figure 2, step 2). Exposed ssDNA is bound by RPA, which is replaced by RAD51 to form RAD51-ssDNA nucleofilaments. These RAD51 nucleofilaments search for homologous sequences present on nearby replicated sister chromatids or homologous chromosomes and invade one or both complementary strands on the donor molecule to form a D-loop or double Holliday junction (dHJ), respectively (Figure 2, step 3 and **4a**). Finally, strand extension of the invaded strand can occur either by synthesis-dependent strand annealing (SDSA) in the case of D-loop formation (**step 3b**) or through canonical DSB repair (DSBR) in the case of dHJ formation. Canonical DSBR occurs at the risk of forming hazardous crossover (CO) products where either sister chromatid or homologous chromosome donor molecules exchange strands of DNA between molecules (Figure 2). SDSA proceeds until there is sufficient sequence homology in the one strand to anneal to the second resected end and continue gap filling and polymerization (Figure 2, step 4b). In canonical DSBR, the risk of forming CO products in turn is a marker of genome instability (West et al., 2016). In the case where a homologous chromosome is used as the template molecule as opposed to a sister chromatid, the heterozygosity of deleterious alleles on one homolog may be lost if that allele is used to repair the DSB containing the healthy allele, leading to a null phenotype (West et al., 2016). When dHJs form, the BLM-TOPOIIIa-RMI1/2 complex can promote convergent migration of the two HJs to produce a hemicatenane structure (Figure 2, step 5a) that can be processed by TOPOIIIa, forming non-CO (nCO) products (West et al., 2016). Alternatively, structure-selective resolvases such as the SLX1/4 and MUS81-EME1 endonucleases can cleave both junctions either symmetrically or asymmetrically to form nCO and CO products, respectively (West et al., 2016). Efforts to limit the risk of CO products aim to favor the DSB repair pathway that leads only to D-loop formation and SDSA. For example, disrupting D-loops before the other overhang of resected DNA anneals with the non-hybridized strand of donor DNA would bias DSBR pathways toward nCO products.

Cells deficient in RECQL5 display a phenotype of genome instability and elevated CO products in the form of SCEs. Hu et al. (2007) discovered that RECQL5 interacts with and disrupts RAD51 nucleofilaments similar to BLM and Sgs1 in yeast, a landmark finding that supported a model of HR where RAD51-dependant pathways are susceptible to CO products and the idea that RECQL5 and BLM are regulators of this pathway in humans. However, the synergistic phenotype of genome instability in *RECQL5^–/–^ BLM^–/–^* double knockouts was the first evidence that these genes may have non-overlapping roles as well. It was later shown *in vivo* that RECQL5 is essential for this disruptive interaction with RAD51 and its ability to form D-loops (Hu et al., 2009).

Bringing these observations together, Olson et al. (2018) proposed a model of HR in which increased levels of RECQL5 reduce repair efficiency in the presence of a dsDNA donor molecule, whereas repair efficiency is significantly increased in the presence of an ssDNA donor. This supports the notion that RAD51 is essential for strand invasion and that by disrupting these nucleofilaments, RECQL5 is limiting the formation of D-loops and subsequent dHJ formation (Paliwal et al., 2014). Given that *RECQL5* gene amplification and deficiency have both been associated with cancer predisposition, it is possible that RECQL5 is required at a suitable level to permit sufficient RAD51-mediated strand invasion for HR repair without an excess of D-loop formation biasing outcomes toward dHJ and CO products (Olson et al., 2018; Wright et al., 2018; Zhu et al., 2018).

### Role of RECQL5 in Replication Stress

**FIGURE 3:**
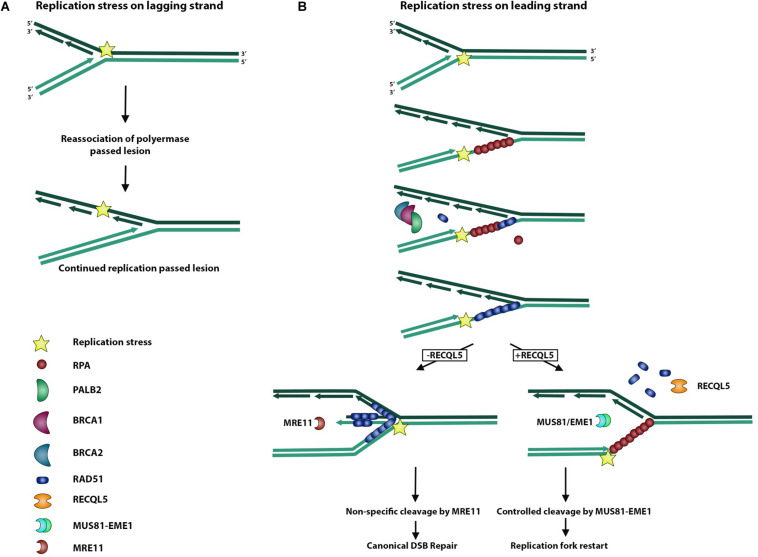
Role of RECQL5 in replication stress response. Replisome can encounter a replication stress-inducing lesion on either a lagging or leading strand. **(A)** When the replisome encounters a lesion on a lagging strand, DNA polymerase is able to bypass the lesion by dissociating from the Okazaki fragment and reassociating to form a new fragment ahead of the lesion. **(B)** Replisome encountering a lesion on a leading strand may lead to replication fork uncoupling, whereby the polymerase is stalled and dissociated from the replication helicase, which continues unwinding DNA and exposing ssDNA. Exposed ssDNA and simultaneous depletion of free RPA serve to activate ATR signaling of the replication stress response and recruit BRCA1/2 and PALB2 to begin exchange of RPA for RAD51. RAD51 actively promotes reversal of the replication fork and formation of a regressed arm whereby newly synthesized DNA strands anneal to each other, allowing for non-specific cleavage by the MRE11 endonuclease and subsequent canonical DSB repair. In the presence of RECQL5, RAD51 is removed from ssDNA, and the MUS81/EME1 endonuclease complex is recruited to allow controlled cleavage and replication fork restart.

During replication, the replisome encounters many stressors that may hinder faithful chromosome duplication (Rickman and Smogorzewska, 2019). This replication stress may slow or even stall the replication fork and activate certain pleiotropic DNA repair genes to form intermediate molecules in an effort to prevent further damage from occurring (Rickman and Smogorzewska, 2019). These replication stress pathways serve to resolve these substructures of DNA, which may arise during replication fork stalling (Saldivar et al., 2017). As a typical by-product of replication fork stalling, the accumulation of exposed ssDNA occurs as RPA is depleted across multiple stalled forks (Toledo et al., 2017). This accumulation and subsequent depletion of free RPA serves to activate ATR kinase and the replication stress response, which serves to recruit DNA repair machinery and stabilize the stalled fork before too much ssDNA is exposed (Figure 3) (Saldivar et al., 2017). Most importantly, it serves to prevent new origins from firing and further RPA depletion and associated ssDNA exposure from leading to global replication fork stalling and replication catastrophe (Toledo et al., 2017). Forks that fail to restart may lead to replication fork collapse and DSBs, activating canonical DSBR pathways (Toledo et al., 2017).

RECQL5 has been implicated in this stress response because of the finding that cells deficient in RECQL5 are hypersensitive to the Topoisomerase I inhibitor, camptothecin, which leads to impaired replication, and experience an exaggerated phenotype of genome instability (Hu et al., 2009). Additionally, RECQL5 associates with the replisome factor, PCNA, and persists at sites of stalled replication forks (Urban et al., 2016). This involvement of RECQL5 in resolving replication stress could, in part, be attributable to its ability to stimulate the endonuclease, FEN1, and coordinate the cleavage events needed for replication fork restart (Speina et al., 2010).

The interaction of RECQL5 with RAD51 also serves an important role in processing stalled replication forks, as RAD51 has a pleiotropic function in both HR and replication stress (Di Marco et al., 2017). Upon replication stress, stalled replication forks accumulate ssDNA, and RAD51 stabilizes this DNA with the support of BRCA2, similar to how RAD51 binds ssDNA on the resected ends of a DSB in DSBR (Figure 3) (Rickman and Smogorzewska, 2019). Electron microscopy studies were performed to study replication fork reversal in the presence and absence of the stabilizing filament, RAD51, its loading partner, BRCA2, and the processing endonuclease, MRE11 (Figure 3). These studies revealed that RAD51 independently promotes replication fork reversal and that RAD51 and BRCA2 together protect against reversed fork degradation by MRE11 (Figure 3) (Mijic et al., 2017). Despite the protective role of RAD51 against MRE11-mediated reversed fork cleavage, overexpression of RAD51 created a phenotype of excessive fork stabilization and impaired replication fork restart, suggesting that an appropriate balance of RAD51-stabilized replication forks is sufficient for replication restart (Mijic et al., 2017). Considering that RECQL5 removes RAD51 filaments in DSBR, Di Marco et al. (2017) examined the role of RECQL5 in replication stress and showed that in addition to removing RAD51 filaments from reversed replication forks, RECQL5 recruits and stimulates the MUS81-EME1 endonuclease complex to promote cleavage and replication restart of difficult-to-replicate regions (Figure 3). Taken together, these findings support a model of RECQL5 in which it balances the intermediate structures in DSBR and the replication stress response.

### Role of RECQL5 in Transcription and Regulating Transcription-Replication Stress

A protein–protein interaction unique to RECQL5 and believed to be critical to its function is that between RECQL5 and the RNA polymerase II (RNAPII) complex (Aygün et al., 2008; Kanagaraj et al., 2010). Cells deficient in RECQL5 display elevated levels of transcription, increased RNAPII-bound chromatin and increased DSBs associated with transcribed loci, suggesting that RECQL5 has more of an inhibitory role in this interaction (Izumikawa et al., 2008; Aygün et al., 2009; Li et al., 2011). Furthermore, RECQL5 loss increased the ratio of RNAPII associated with promoter-proximal regions relative to the gene body of a subset of over 5000 genes examined, whereas overexpression reversed this ratio (Saponaro et al., 2014). However, there was no change in overall mRNA produced, suggesting that transcription elongation rate was affected, as opposed to transcription initiation (Saponaro et al., 2014). For 80% of the transcribed genes in a genome-wide assay, Saponaro et al. (2014) created an *in vivo* model to synchronize transcript cycles and measure the elongation rate of individual genes and showed that depletion of RECQL5 significantly increased this value whereas overexpression reduced it. In the absence of RECQL5, sites with elevated transcript elongation were enriched for DSB breaks. Together, these findings suggest that RECQL5 is an inhibitory RNAPII elongation factor and that deficiencies in RECQL5 lead to increased rates of RNAPII-mediated transcript elongation, higher levels of RNAPII pausing or arrest and overall transcription-induced genome instability. This form of transcription-associated genome instability appears also to be associated with replication, since Li et al. (2018). showed that many of the DSBs in this model accumulate during S-phase and associate with RNA-transcribed loci. This phenotype was relieved in the presence of a transcription inhibitor, further supporting the association of replication and transcription machinery driving DSBs and genome instability (Li et al., 2011). Together these findings support a model of transcription-associated genome instability where RECQL5 is limiting the collision of transcription and replication machinery by slowing the elongation rate of transcription.

Another source of transcription-associated genome instability is the formation of R-loop structures at sites of active transcription during replication. The formation of ssDNA from negative supercoiling behind transcription allows RNA invasion, forming an R-loop and making it difficult for replication machinery to continue (Li et al., 2018). RECQL5-bound RNAPII was shown to stimulate conjugation of SUMO2 to the replicative factor, PCNA, another one of its binding partners (Li et al., 2018). Conjugated SUMO2-PCNA is capable of interacting with the histone chaperone protein, CAF1, and depositing repressing histone marks in a CAF1-dependant manner, thereby reducing chromatin accessibility and effectively dislodging RNAPII from DNA (Li et al., 2018). This was confirmed by showing that cells deficient in RECQL5 are transcription replication conflict (TRC) and DSB prone and that overexpressing SUMO2-PCNA or CAF1 rescued this phenotype (Li et al., 2018). Additionally, RECQL5 was shown to mediate replication fork restart at the sites of stalled replication forks near R-loops by limiting RAD51-mediated replication fork reversal and recruiting the MUS81-EME1 endonuclease complex for appropriate processing of stalled replication (Chappidi et al., 2019).

These findings support a model of RECQL5 that intimately relates transcription to replication and serves to limit TRCs. There is evidence that it does so both proactively by either inhibiting transcript elongation near sites of replication or remodeling chromatin to dislodge RNAPII from DNA and retroactively by limiting RAD51-mediated replication fork reversal and promoting MUS81-EME1 cleavage and replication fork restart (Di Marco et al., 2017; Li et al., 2018; Chappidi et al., 2019).

## Discussion

It is clear that RECQL5 serves as an important regulator of DNA repair intermediate structures that may arise during DNA damage, replication stress, and transcriptional stress. This essential regulatory role of RECQL5 is further highlighted by the observed elevated RECQL5 expression and gene amplification in urothelial carcinoma of the bladder and in breast cancers (Chen et al., 2015; Arora et al., 2016; Patterson et al., 2016). However, the nature of DNA lesions that are preferentially repaired using RECQL5, the choice of RECQL5 over alternative RecQ helicases for the repair of various DNA lesions, and the role of expression levels in such choices remain to be elucidated. The finding of significant cancer predisposition in mice models deficient in RECQL5 supports the hypothesis that perturbation of RECQL5 levels in either direction can contribute to oncogenesis (Hu et al., 2007). Yet it remains unclear to what degree RECQL5 is the only factor regulating these processes and how RECQL5 contributes to oncogenesis or provides a backup function to other essential DNA repair genes. There is evidence of some overlapping function, specifically with other RecQ helicases. For example, in comparison to BLM, RECQL5 shares a similar phenotype of genome instability, but there is sufficient evidence that RECQL5 suppresses SCEs and DSBs even in the presence of BLM (Hu et al., 2005). Shared protein–protein interactions between RECQL5 and BLM, such as with RAD51, likely correspond to overlapping functions, whereas interactions unique to RECQL5, such as that with RNAPII, may provide useful insight into the unique functions of RECQL5 (Li et al., 2011; Paliwal et al., 2014).

The larger body of research on other RecQ helicases supports further studies of RECQL5 in parallel with other RecQ helicases. Given that loss of RECQL5 increases SCEs, it will be of interest to map the location of such events, as it was shown that BLM preferentially prevents SCE events near transcribed genes and G-quadruplex motifs (van Wietmarschen et al., 2018). There may be specific motifs or substructures of DNA that RECQL5 preferentially localizes to and protects against genome instability. Such studies will help uncover the role of RECQL5 in the maintenance of genome stability and might provide clues about its involvement in oncogenesis.

## Author Contributions

ZH wrote the first draft of the manuscript. ZH and PL contributed to manuscript revision and approved the submitted version.

## Conflict of Interest

The authors declare that the research was conducted in the absence of any commercial or financial relationships that could be construed as a potential conflict of interest.

## Abbreviations

CO product: crossover product
dHJ: double Holliday junction
DSB: double-strand break
nCO product: non-crossover product
RQC: RecQ C-terminal
SCE: sister chromatid exchange event
TRC: transcription replication conflict.
